# Left Lung Torsion: Complication of Lobar Resection for an Early Stage Lung Adenocarcinoma

**DOI:** 10.1155/2016/9240636

**Published:** 2016-05-17

**Authors:** Wissam Mansour, Elias Moussaly, Ali Abou Yassine, John Nabagiez, Rabih Maroun

**Affiliations:** ^1^Department of Internal Medicine, Northwell Health Staten Island University Hospital, 475 Seaview Avenue, Staten Island, NY 10305, USA; ^2^Department of Pulmonology and Critical Care Medicine, Northwell Health Staten Island University Hospital, 475 Seaview Avenue, Staten Island, NY 10305, USA; ^3^Department of Cardiothoracic Surgery, Northwell Health Staten Island University Hospital, 475 Seaview Avenue, Staten Island, NY 10305, USA

## Abstract

Lobar torsion is a fatal but fortunately rare occurrence following lung resection. Early clinical signs and radiographic features may be nonspecific resulting in diagnostic delay. A high index of suspicion is vital for early diagnosis and intervention to avoid further parenchymal necrosis and deadly gangrene. We report a case of left lower lobe torsion in a 76-year-old female following elective upper lobectomy for underlying lung adenocarcinoma. Diagnosis was made following highly suggestive radiographic findings prompting bronchoscopy and revision thoracotomy. An emergency detorsion failed to restore lung viability and was followed by completion pneumonectomy. The patient recovered and was discharged on the seventh postoperative day.

## 1. Introduction

Lobar torsion is a very rare but potentially fatal postlobectomy complication. It represents a diagnostic dilemma in the early postoperative period and a high index of suspicion is crucial for lung salvage. Torsion most commonly involves the middle lobe following ipsilateral lobectomy with only few cases describing left sided involvement. We will present a case of left lower lobe torsion, followed by a review of the medical literature on this topic.

## 2. Case Presentation

**Figure 1:**
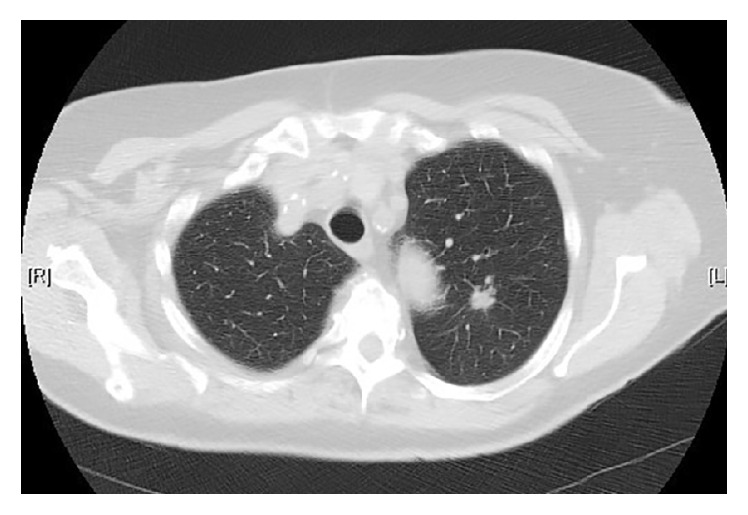
CT of the chest parenchymal view demonstrating a 1.2 cm spiculated left upper lobe nodule.

Our patient is a 76-year-old lady with chronic obstructive pulmonary disease, peripheral vascular disease, and smoking as past medical history, presenting with 2 months of a nonproductive cough. The patient was found to have a left upper lobe nodule on a plain chest radiograph and further evaluation with a computed tomography (CT) of the chest delineating a 1.2 cm spiculated nodule (Figure 1). Following a PET-CT that showed pathologic FDG uptake of the lesion, the patient underwent elective left thoracotomy with upper lobe lobectomy and mediastinal lymph node resection. Pathology showed a 1.5 × 0.6 cm papillary predominant adenocarcinoma with no lymph node metastases.

**Figure 2:**
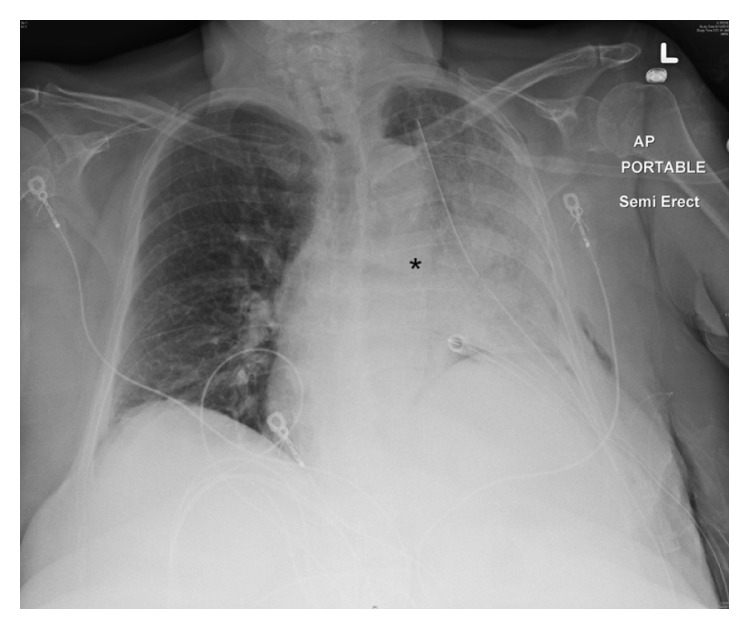
Portable chest radiograph obtained on postop day-2 showing left lung opacification with bronchial cutoff (black asterisk).

**Figure 3:**
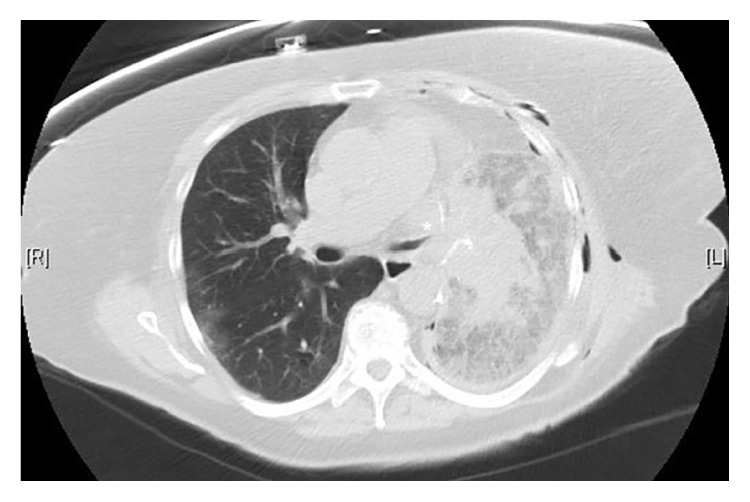
CT of the chest parenchymal view demonstrating left lobar consolidation with ground glass attenuation and proximal bronchial tapering (white asterisk).

**Figure 4:**
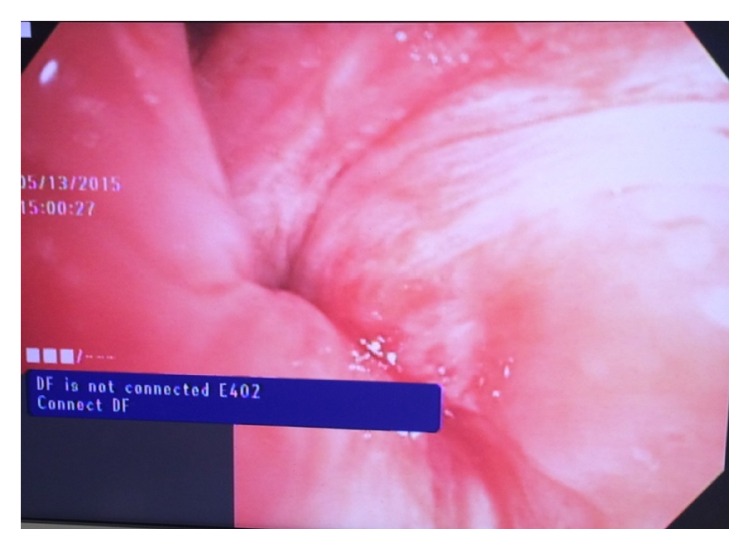
Flexible bronchoscopy showing tight left lower lobe orifice.

**Figure 5:**
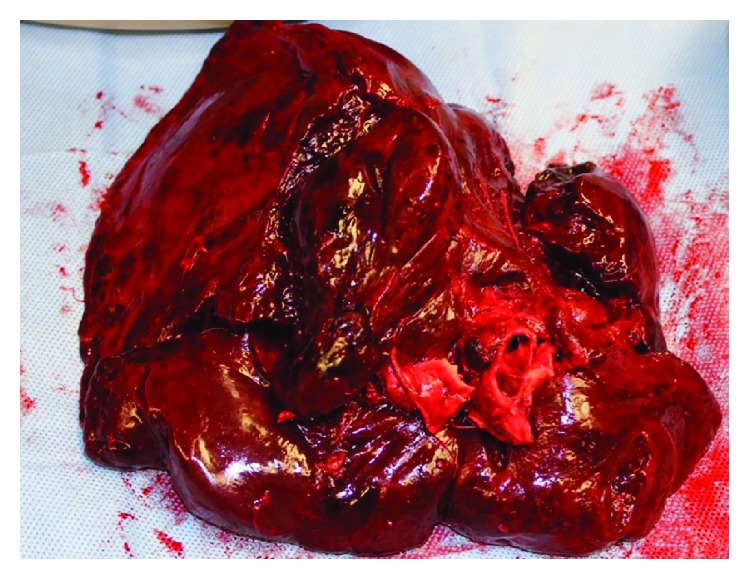
Gross appearance of left lower lobe following left pneumonectomy completion.

The immediate postoperative course was uncomplicated and the patient was transferred to the cardiothoracic unit for observation. Follow-up chest radiography on postoperative day-1 showed a small left lower lobe opacity with the patient complaining of mild pain at the surgical site. On postoperative day-2 the patient developed atrial fibrillation with rapid ventricular rate associated with hemoptysis and worsening left sided pleuritic chest pain. Plain chest radiography showed worsening left lung opacity with bronchial cutoff (Figure 2). A CT of the chest revealed diffuse ground glass attenuation of the left lower lobe with proximal bronchial obliteration (Figure 3). The patient underwent elective intubation to undergo flexible bronchoscopy that revealed a tight and obstructed orifice of the left lower lobe (Figure 4). She was brought to the operating room emergently for high suspicion of lobar torsion, and a left thoracotomy showed a dark purple appearing lung (Figure 5). Following detorsion the lung did not appear viable; however considering the patient's baseline marginal FEV1, 24 hours was given for the lung to recover. The patient showed no clinical improvement and underwent completion of left lung pneumonectomy the next day. Postoperative recovery was uneventful and patient was discharged after 7 days. The patient was seen in the outpatient office 4 months following discharge reporting no complications.

## 3. Discussion

Lung torsion is the rotation of a pulmonary lobe around the hilar pedicle resulting in bronchovascular compromise. It is a rare event seen mainly in the postoperative setting following thoracic surgery [1, 2]. Fewer cases described its occurrence spontaneously or secondary to blunt trauma and pleural effusion [1, 3, 4]. The incidence of postoperative torsion varied with reported series ranging from 0.089 to 0.2%, predominantly involving the middle lobe following ipsilateral lobectomy [1, 5]. To our knowledge four cases have been reported in the English literature describing postlobectomy left lower lobe torsion [6–9].

The underlying pathophysiology remains controversial with reports delineating few predisposing factors [1, 2, 10]: (i) increased mobility secondary to inferior pulmonary ligament transection [11, 12]; (ii) complete interlobar fissures resulting in loss of parenchymal bridge between contiguous lobes; (iii) an easily displaceable milieu of air or fluid in cases of pneumothorax or pleural effusion [4]; (iv) a heavy compact airless lobe in cases of atelectasis and consolidations; (v) a relatively longer left lobar pedicle [2, 11]; (vi) pedicle skeletonisation during hilar lymph node removal [13].

The clinical picture of lung torsion varies depending on whether the torsion is partial or complete. Partial torsion has an insidious course presenting with partial collapse or obstructive pneumonia [2]. On the other hand complete torsion predisposes to a more dramatic presentation characterized by fever, tachycardia, hemoptysis, chest pain, hypoxemia, and loss of breath sounds at the affected side [1, 2].

Recognition of early imaging features is vital for prompt diagnosis and management. Plain radiography can raise early suspicion for postoperative torsion if certain findings are delineated including inappropriate hilar displacement away from the collapsed lobe, collapsed lobe in an unusual location, ipsilateral lobe opacification following surgery, and bronchial cutoff (Figure 2) [10]. CT findings are more specific including lobar consolidation with ground glass attenuation and interlobular septal thickening associated with tapered obliteration of the proximal bronchus and artery of the involved lobe (Figure 3) [2, 14]. Direct visualization through bronchoscopy can be diagnostic, revealing an occluded bronchus of the involved segment without evidence of a mucous plug or endobronchial lesion (Figure 4).

Following suspicion of torsion, an emergent surgical intervention is vital. The optimal type of intervention remains controversial depending on the lung status visualized by thoracotomy. If the involved lobe demonstrates viability, detorsion may offer the possibility for lung salvage [15]. However few reports noted rapid deterioration of patients after detorsion in a simulation to ischemia-reperfusion injury when intervention was delayed [16]. Considering the rapid progression of lung gangrene following torsion the option for simple detorsion should only be advocated in patients with early reintervention [17]. In cases where intervention is delayed lobectomy remains the best option.

Prophylactic lung fixation has been proposed as a successful method to prevent lung torsion using various surgical techniques including parenchymal suturing, adhesive membranes, and pleural flaps [11, 13, 18].

In regard to our patient, she was at higher risk for a very rare complication of left lower lobe torsion secondary to surgical inferior pulmonary ligament transection and pedicle skeletonisation, a complete left longitudinal fissure, and a longer left lobar pedicle. Initially she presented with atrial arrhythmia and hemoptysis with no significant hypoxemia or respiratory distress. Early diagnosis in the first 24 hours was made possible with highly suggestive radiographic features; however the lung did not appear viable at the time of intervention. Lung detorsion performed within 24 hours did not result in clinical deterioration; however lung viability also did not improve and the patient eventually underwent complete pneumonectomy.

Lung torsion remains a fatal but fortunately rare complication after lobectomy. A low threshold should be present in at risk patients for prompt investigation and intervention that is vital for lung salvage. Patients with multiple predisposing factors should be considered for prophylactic lung fixation.
